# Extracellular vesicles from hair follicle-derived mesenchymal stromal cells: isolation, characterization and therapeutic potential for chronic wound healing

**DOI:** 10.1186/s13287-022-02824-0

**Published:** 2022-04-08

**Authors:** Kevin Las Heras, Félix Royo, Clara Garcia-Vallicrosa, Manoli Igartua, Edorta Santos-Vizcaino, Juan M. Falcon-Perez, Rosa Maria Hernandez

**Affiliations:** 1 NanoBioCel Group, Laboratory of Pharmaceutics, School of Pharmacy (UPV/EHU), 01006 Vitoria-Gasteiz, Spain; 2 Bioaraba, NanoBioCel Research Group, Vitoria-Gasteiz, Spain; 3 Center for Cooperative Research in Biosciences (CIC bioGUNE), Basque Research and Technology Alliance (BRTA), Exosomes Laboratory, 48160 Derio, Spain; 4 Centro de Investigación Biomédica en Red de Enfermedades Hepáticas Y Digestivas (CIBERehd), 28029 Madrid, Spain; 5 Biomedical Research Networking Centre in Bioengineering, Biomaterials and Nanomedicine (CIBER-BBN), 28029 Madrid, Spain; 6 IKERBASQUE, Basque Foundation for Science, 48013 Bilbao, Spain

**Keywords:** Extracellular vesicles, Exosomes, Microvesicles, Mesenchymal stromal cells, Chronic wounds, Skin wounds, Hair follicle, Diabetic ulcers

## Abstract

**Background:**

Mesenchymal stromal cells (MSCs) and their extracellular vesicles (MSC-EVs) have demonstrated to elicit immunomodulatory and pro-regenerative properties that are beneficial for the treatment of chronic wounds. Thanks to different mediators, MSC-EVs have shown to play an important role in the proliferation, migration and cell survival of different skin cell populations. However, there is still a big bid to achieve the most effective, suitable and available source of MSC-EVs.

**Methods:**

We isolated, characterized and compared medium-large EVs (m-lEVs) and small EVs (sEVs) obtained from hair follicle-derived MSCs (HF-MSCs) against the gold standard in regenerative medicine, EVs isolated from adipose tissue-derived MSCs (AT-MSCs).

**Results:**

We demonstrated that HF-EVs, as well as AT-EVs, expressed typical MSC-EVs markers (CD9, CD44, CD63, CD81 and CD105) among other different functional markers. We showed that both cell types were able to increase human dermal fibroblasts (HDFs) proliferation and migration. Moreover, both MSC-EVs were able to increase angiogenesis in human umbilical vein endothelial cells (HUVECs) and protect HDFs exposed to a hyperglycemic environment from oxidative stress and cytotoxicity.

**Conclusions:**

Taken together, HF-EVs demonstrated to exhibit comparable potential to that of AT-EVs as promising candidates in the treatment of chronic wounds.

**Supplementary Information:**

The online version contains supplementary material available at 10.1186/s13287-022-02824-0.

## Background

Hair follicles (HFs) are skin appendages originated by the interactions of ectodermal-mesodermal cells in the early embryogenesis. HFs present a complex structure with a rich mixture of cell populations. It has been described that HFs contain different pools of stem cells—melanocyte, epithelial and mesenchymal stromal cells (MSCs)—that regulate hair growth, maintaining skin homeostasis and self-renewing processes continuously [1–3]. In the past decade, there has been an increasing interest in these hair follicle derived MSCs (HF-MSCs) due to their numerous advantages over other sources of MSCs—abundant availability of HFs that do not undergo functional and molecular changes in the human body and an easy collection not affected by gender or age—[2, 4, 5]. Furthermore, their role in skin homeostasis, hair growth and their pro-regenerative capacity are making these cells become one of the most suitable candidates for cell-based wound healing therapies [6–10]. Indeed, it has been observed that the direct administration of HF-MSCs can accelerate wound healing both in vitro and in vivo in diabetic mice models [11].

However, despite its demonstrated efficacy and safety, the use of cell therapy for chronic wound management have found important constraints through the harsh regulatory pathways and elevated production costs that impede its widespread use [7, 8, 12]. As a result, numerous researchers have studied the paracrine signaling of MSCs, which has been described as the main responsible for the therapeutic potential of these cells [13–15]. Among the bioactive cues that drive regenerative effects, extracellular vesicles (EVs) have gained special attention. In the form of membrane-surrounded units, EVs shuttle the messaging cargos of RNAs, DNAs and proteins to mediate intercellular communications [16]. Similarly to MSC-based therapies, MSCs-derived EVs (MSC-EVs) have demonstrated to elicit a wide variety of therapeutic effects in tissue repair and wound regeneration—immunomodulatory, proliferative, anti-apoptotic, pro-angiogenic effects etc.—[17–19]. Furthermore, EVs are more easily handled, characterized and stored than MSCs. In this regard, EVs-based therapies can be better standardized than cell-based therapies. Moreover, these therapies can be commercialized under a softer regulation pathway than cell-based therapies, being considered “biological medicines” [20].

**Fig. 1:**
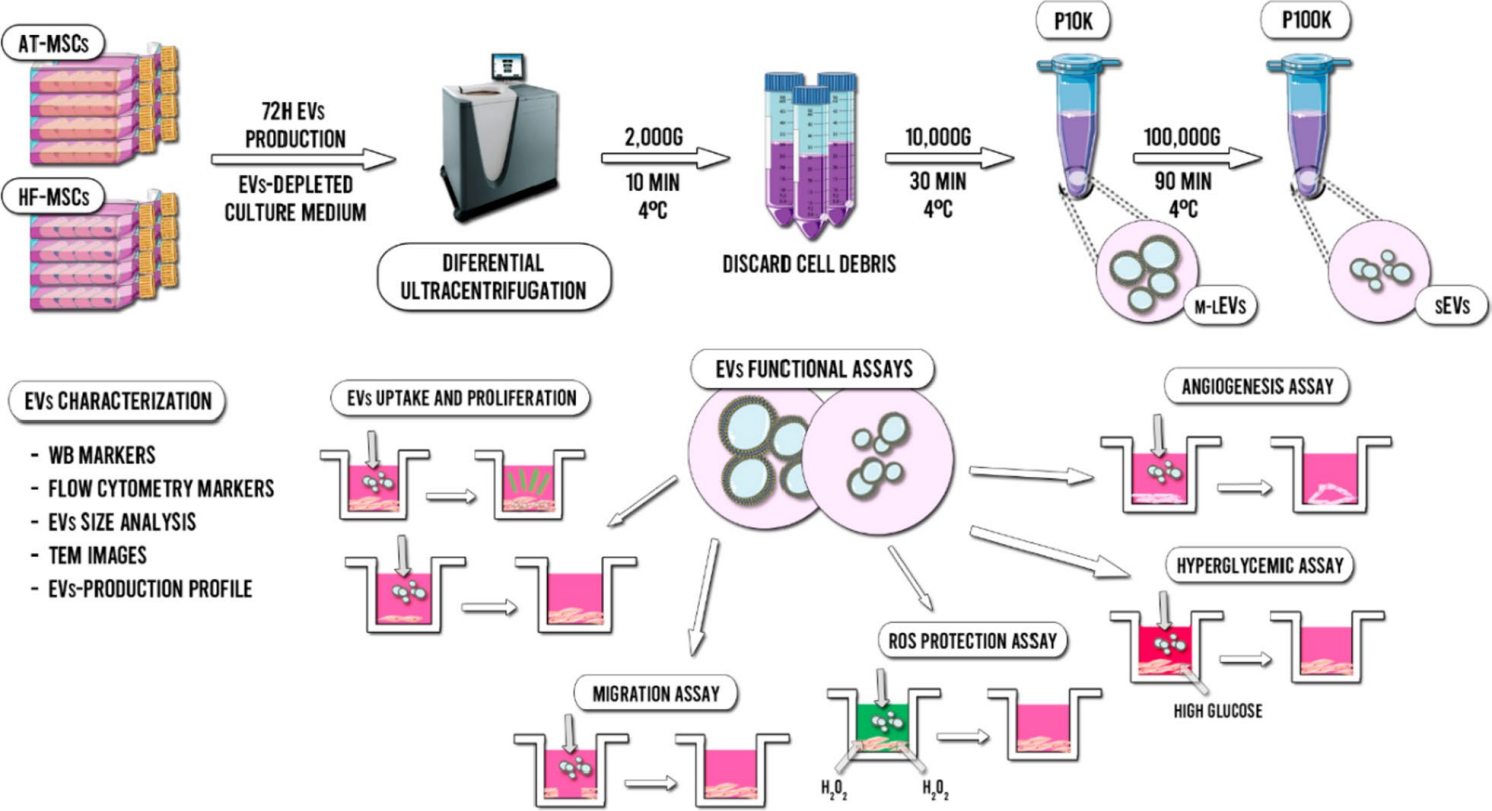
Scheme of the isolation method, characterization and functional assays of HF-EVs and AT-EVs

In this study, we report a complete characterization and functional comparison of EVs isolated from HF-MSCs (HF-EVs) against the gold standard in regenerative medicine, EVs derived from adipose tissue MSCs (AT-EVs) (Fig. 1). We have analyzed the MSC-EVs production profile, size, morphology and marker expression. Furthermore, we have compared the cell uptake and tested their potential in human dermal fibroblasts (HDFs)—one of the most important cell types involved in the wound healing process—and in human umbilical vein endothelial cells (HUVECs) to study the vascular behaviour of wounds. Following the MISEV 2018 guidelines [21], we have analyzed not only of the small EVs (sEVs) released by these cells, but also the medium-large EVs (m-lEVs) that are sometimes discarded, despite their demonstrated potential for regenerative medicine applications.

## Materials and methods

### EVs isolation and characterization

#### Cell culture conditions

HF-MSCs were isolated and characterized from HFs as previously described [22] and further cultured in Dulbecco’s modified Eagle’s medium (DMEM 49166-029, Gibco) supplemented with 10% of fetal bovine serum (FBS, Gibco) and 1% (v/v) penicillin/streptomycin (P/S, Gibco). AT-MSCs (ATCC® PCS-500–011™) were also cultured under the same conditions. Adult Human Dermal Fibroblasts (HDFs, ATCC® PCS-201-012™) were cultured in fibroblasts basal medium (ATCC PCS-201-030™) adding the fibroblasts growth kit-low serum (ATCC® PCS-201-041™) and 1% (v/v) penicillin/streptomycin (P/S, Gibco). Incubating conditions were 37ºC in a 5% CO_2_ atmosphere. Human umbilical vein endothelial cells (HUVECs, Lonza® C2517A) were cultured in the EGM™-2 Endothelial Cell Growth Medium-2 BulletKit™ (Lonza® CC-3162) with 1% (v/v) penicillin/streptomycin (P/S, Gibco). For EVs production, DMEM medium was used with 10% of EV-depleted FBS—by overnight ultracentrifugation 100,000×*g* (Beckman Coulter, Optima L-100XP)—. HF-MSCs and AT-MSCs were used at passages from 5 to 9 for EVs isolation. HDFs were used at passages 3–7. HUVECs were used at passages 3–7.

#### EVs isolation

All relevant data regarding the production, isolation and experimental section have been submitted to the EV-TRACK knowledgebase (EV-TRACK ID: EV210337) [23]. EVs were isolated and purified from the supernatant of HF-MSCs and AT-MSCs. At 70–80% of confluency, cells were washed thrice with PBS and the culture medium was replaced with EVs-depleted DMEM. After 72 h of production, the culture medium was collected and new medium was added. After three collections, cells were trypsinized, counted and further re-cultured. The collected medium was first centrifuged at 2000×*g* for 10 min at 4ºC to discard cell debris and then, freeze at -80ºC until the isolation and purification was given. All further centrifugation processes were performed at 4ºC. In brief, thawed culture supernatants were differentially centrifuged at 10,000×*g* for 30 min to obtain m-lEVs—pellet 10 K (P10K)—and the resulting supernatants at 100,000×*g* for 90 min—pellet 100 K (P100K)—to obtain sEVs. To reduce contaminating proteins, all pellets (P10K and P100K) were re-suspended in ice-cold PBS and ultracentrifuged again. Finally, both pellets were immediately re-suspended in 120 µL of ice-cold PBS and freeze at − 80ºC until use.

#### Nanoparticle tracking analysis

Particle concentration and size distribution within EV preparations was analyzed using the nanoparticle-tracking analysis (NTA), by measuring the rate of Brownian motion in a NanoSight LM10 system (Malvern Panalytical, Malvern, UK). The system was equipped with a fast video-capture and particle-tracking software. NTA post-acquisition settings were the same for all measurements. Each video was analyzed to give the mean, mode, and median vesicle size, as well as an estimate of the concentration. For measurement, original EVs suspension were diluted 1:100 with PBS and a volume of 500 µL were loaded on the camera, and 3 consecutive video recording of 40 s each were taken for every sample quantified.

#### Western blotting

PBS-resuspended EVs or cell lysates were mixed with 4 × NuPAGE LDS Sample Buffer (Thermo Fisher Scientific, Waltham, MA, USA). The samples were incubated for 5 min at 37 °C, 65 °C, and 95 °C, and separated on 4–12% precast gels (from Thermo Fisher Scientific, Waltham, MA, USA), at a concentration of 5 µg/lane (Bradford determination). The proteins were transferred into PVDF membranes with the iBLOT2 system (Thermo Fisher Scientific, Waltham, MA, USA). Antibodies employed were: mouse monoclonal antibody against CD9 #209,302 (R&D), Grp78 #40 (BD, East Rutherford, NJ, USA), CD13 #3D8 (Santa Cruz Biotechnology, Dallas, Tx, USA), EEA1 #14 (BD, East Rutherford, NJ, USA), CD63 #H5C6 (DSHB, Iowa City, IA, USA), LAMP1 #H4A3 (DSHB, Iowa City, IA, USA), alpha tubulin 1 #DM1A (Santa Cruz Biotechnology, Dallas, Tx, USA), CD81 #JS81 (BD, East Rutherford, NJ, USA), rabbit antibody against COX IV #4850 (Cell Signaling Technologies, Danvers, Ma, USA). All the primary antibodies were diluted 1:1000. Horseradish peroxidase (HRP)-conjugated secondary antibodies anti-mouse, rabbit and goat, were purchased from Jackson ImmunoResearch Lab, (West grove, PA, USA) to ensure minimal cross-reactivity across species.

#### Bead-based multiplex flow cytometry assay

All isolated EVs were subjected to a surface-marker characterization by using a flow cytometry bead-based multiplex analysis (MACSPlex Exosome Kit, human, Miltenyi Biotec. 130-108-813). Samples were processed according to manufacturer’s protocol. Briefly, 2 µg of EVs were mixed with 120 µL of manufacturer’s buffer and then, 15 µL of the MACSPlex Exosome Capture Beads were added. Then, 15 µL of the detection antibody cocktail—5 µL of each MACSPlex exosome detection reagent CD9, CD63, CD81—were added. After that, samples were incubated 1 h at room temperature, protected from the light, on rotation—450 rpm. Next, 500 µL of MACSPlex buffer were added and samples were centrifuged at 3000×*g* for 5 min. Subsequently, 500 µL of supernatant were discarded and another 500 µL of buffer were added. Samples were incubated for 15 min at room temperature on rotation, protected from the light and then centrifuged 5 min at 3000×*g*. Finally, 500 µL of supernatants were discarded and approximately 150 µL of samples were re-suspended and used for the analysis. The flow cytometry analysis was performed using the MACSQuant® Analyzer 10 (Miltenyi Biotec) and results were processed with the MACSQuant Analyzer 10 software (Miltenyi Biotec). The 39 single bead populations were gated to determine the APC signal intensity on each bead population and the median fluorescence intensity (MFI) for each capture bead was measured. For each population, background was corrected by subtracting the respective MFI values from non-EVs controls that were treated exactly like the EVs-samples. Furthermore, values of the corresponding isotype control were also subtracted. Only positive markers are shown in the graphics.

#### Cryo-electron microscopy (cryo-EM)

EVs (3–5 µL) were spotted on glow-discharged lacey grids and cryo-fixed by plunge freezing at -180 °C in liquid ethane with a Vibrobot (FEI, The Netherlands). Grids were observed with a JEM-2200FS/CR TEM (JEOL, Japan), operating at 200 kV. Image measurements were performed with Image J software and between 80 and 120 single EVs were measured on each group for size and protein-decoration analysis.

#### CD63 immunostaining

Cells were grown onto 12 mm glass coverslips and fixed with 4% formaldehyde, and permeabilized with 0.1% saponin and 0.1% BSA in PBS. Incubation with primary and secondary antibodies were performed in a humid camera for 1 h each. Following two-10 min washes in PBS, cells were mounting with Fluoromount-G™ Mounting Medium with 2-(4-amidinophenyl)-1 h-indole-6-carboxamidine (DAPI) (Thermo Fisher Scientific, Waltham, MA, USA) for nuclei staining. Imaging of the slides were taken in a Leica SP8 confocal microscope.

### EVs uptake and proliferation by HDFs

#### EVs staining

In order to stain EVs, Vybrant™ DiO Cell-Labeling Solution (Invitrogen, Cat. V22886, Thermo Fisher Scientific, Waltham, MA, USA) was used. Briefly, cultured HF-MSCs and AT-MSCs were washed with PBS and 50 µL of dye in 10 mL of culture medium were added. Cells were incubated for 30 min and after that, washed again thrice with PBS. Finally, EVs-depleted medium was added and a 72 h labeled-EVs production was obtained as previously described. As negative control, the same exact process was performed in flask without cells, to determine the possible carry-over of fluorescence by the culture medium.

#### EVs uptake by HDFs and flow cytometry

To perform capture experiments, 500,000 HDFs per well were seeded in 6 well plates and 12 mm slides, and incubated overnight with MSC-EVs stained as described above diluted in 2 mL of medium. Slides were fixed and directly observed in a Leica SP8 confocal microscopy, while HDFs in wells were washed twice with PBS and detached from the plate using TrypLE Select (1X) (Gibco, 12604013) and directly observed in a Cytoflex flow cytometer (Beckman Coulter Inc, Chicago, Il, USA), and data analysis was performed using the CytExpert v2.4 software (Beckman Coulter Inc, Chicago, IL, USA).

#### Proliferation assay

HDFs were seeded in 96 well plates—5000 cells/well—in 100 µL of complete medium. After 8 h of incubation, cells were washed with PBS and then cultured overnight with serum-free medium. After that, cells were washed with PBS and EVs were administered—1.5 × 10^11^ EVs/mL of P10K and 2 × 10^11^ and 4 × 10^11^ EVs/mL of P100K—in 100 µL of 1:5 complete medium in serum-free medium and incubated for 24 h. Complete medium was used as positive control. After that, cells were washed with PBS and 100 µL of CCK8 (Merck, Cat: 96992) 1:10 diluted in culture medium were added. After 4 h of incubation, absorbance was read with a plate reader (Infinite® 200 PRO series, TecanTrading AG, Männedorf, Switzerland) at 450 nm, using 650 nm as the reference wavelength.

### Scratch assay

To evaluate whether EVs can promote migration of HDFs a scratch assay was performed. HDFs were seeded—70 µL at a density of 5 × 10^5^ cells/mL—in each side of the 2 well IBIDI culture inserts® in 24-well plates (IBIDI, Cat. 80209) and subsequently were incubated for 8 h. Secondly, the culture medium was aspired and serum-free medium was added in order to minimize the cell proliferation. After an overnight incubation, culture inserts were extracted and the scratch was performed. Then, HDFs were washed with PBS, EVs were administered—2.5 × 10^9^ EVs/mL of P10K and 1 × 10^10^ EVs/mL of P100K—in 300 µL of serum-free medium and cells were incubated for 48 h. At pre-defined time-points—0 h, 6 h, 12 h, 24 h, 36 h and 48 h—images of the closing area were taken by an optical microscope (Nikon, Japan). The percentage of migration in the area between wound edges was calculated by ImageJ software (National Institutes of Health, MA, USA).

### Tube formation assay

To perform the tube formation assay 15 µL of Matrigel® matrix (Corning®, Cat. 356231) were added into the µ-Plate angiogenesis 96 well plates (IBIDI, Cat. 89646) and let polymerize for 30 min at 37 °C. HUVECs were seeded at a density of 4.5 × 10^5^ cells/mL in 30 µL of complete medium, 15 µL of PBS and 25 µL of EVs—2 × 10^11^ EVs/mL of P10K and 3.5 × 10^11^ EVs/mL of P100K—in the matrigel bed. Medium with non-EVs control batches processed equally to the EVs batches was used as control. Complete medium was used as positive control. Following an incubation of 24 h, each well was photographed by an optical microscope (Nikon, Japan). After that, cells where stained with calcein AM (Invitrogen, “LIVE/DEAD™ Viability/Cytotoxicity Kit, for mammalian cells” Cat. L3224) and each well was photographed by a Nikon epi-fluorescence microscope equipped with a DSD2 confocal modulus (Nikon, Japan). The observed tubes were analyzed by using the ImageJ software.

### Cell survival under hyperglycaemia assay

The effect of EVs on cell survival under a hyperglycaemic environment was studied in HDFs. Briefly, 10,000 cells were cultured in 96 well plates for 24 h. After that, HDFs were pre-treated with EVs—1 × 10^11^ EVs/mL of P10K and 3 × 10^11^ EVs/mL of P100K—for 6 h, rinsed with PBS and exposed to hyperglycaemic conditions of 150 mM glucose in HDFs serum-free culture medium. Cells pre-treated with non-EVs control batches processed equally to the EVs batches were used as control. Cells pre-treated with complete medium were used as a positive control. Results were normalized against cells grown under non-hyperglycaemic conditions. The metabolic activity was observed at 24 h, 48 h and 72 h by CCK8 assay as described earlier. Cell viability at 72 h was also observed with calcein AM/Ethidium homodimer (Invitrogen, “LIVE/DEAD™ Viability/Cytotoxicity Kit, for mammalian cells” Cat. L3224) and each well was photographed by a Nikon epi-fluorescence microscope equipped with a DSD2 confocal modulus (Nikon, Japan).

### Reactive oxygen species (ROS) protection assay

Cell viability of HDFs under a ROS environment was studied in the protection assay. In brief, HDFs—1 × 10^5^ cells/well—were seeded in a 96-well plate and allowed to adhere. After 24 h, cells were washed with PBS and the EVs pre-treatment—1 × 10^11^ EVs/mL of P10K and 3 × 10^11^ EVs/mL of P100K—was added for 2 h and 6 h respectively. After that, cells were washed and then incubated for 30 min under a ROS environment—100 µL of serum free medium with 15 mM of H_2_O_2—_. Then, cells were washed with PBS and incubated overnight in serum free medium to minimize the proliferation of the surviving cells. Finally, cells were washed with PBS and 100 µL of CCK8, 1:10 diluted in serum free culture medium, were added. After 4 h of incubation, the absorbance was read at 450 nm, using 650 nm as the reference wavelength. An alternative co-culture ROS assay was performed as follows. HDFs—1 × 10^5^ cells/well—were seeded in a 96-well plate and allowed to adhere. After 24 h, cells were washed with PBS and then EVs were added in 100 µL of serum free medium with 625 µM of H_2_O_2_ for 4 h. After that, cells were washed with PBS and 100 µL of CCK8, 1:10 diluted in serum free culture medium, were added. After 4 h of incubation, absorbance was read. For all assays, medium with non-EVs control batches processed equally to the EVs batches was used as control and complete medium was used as positive control.

### Statistical analysis

Results are expressed as the mean ± standard deviation. When normally distributed, results were analyzed through Student’s two-tailed *t* test, to compare between two independent groups, or through a one-way ANOVA test for multiple comparisons. Based on the Levene test for the homogeneity of variances, Bonferroni or Tamhane post-hoc analyses were applied. In contrast, Mann–Whitney’s non-parametric analysis was applied for non-normally distributed data. All the statistical computations were performed using SPSS 25.0 (SPSS®, Inc., Chicago, IL, USA).

## Results

### EVs isolation and characterization

**Fig. 2:**
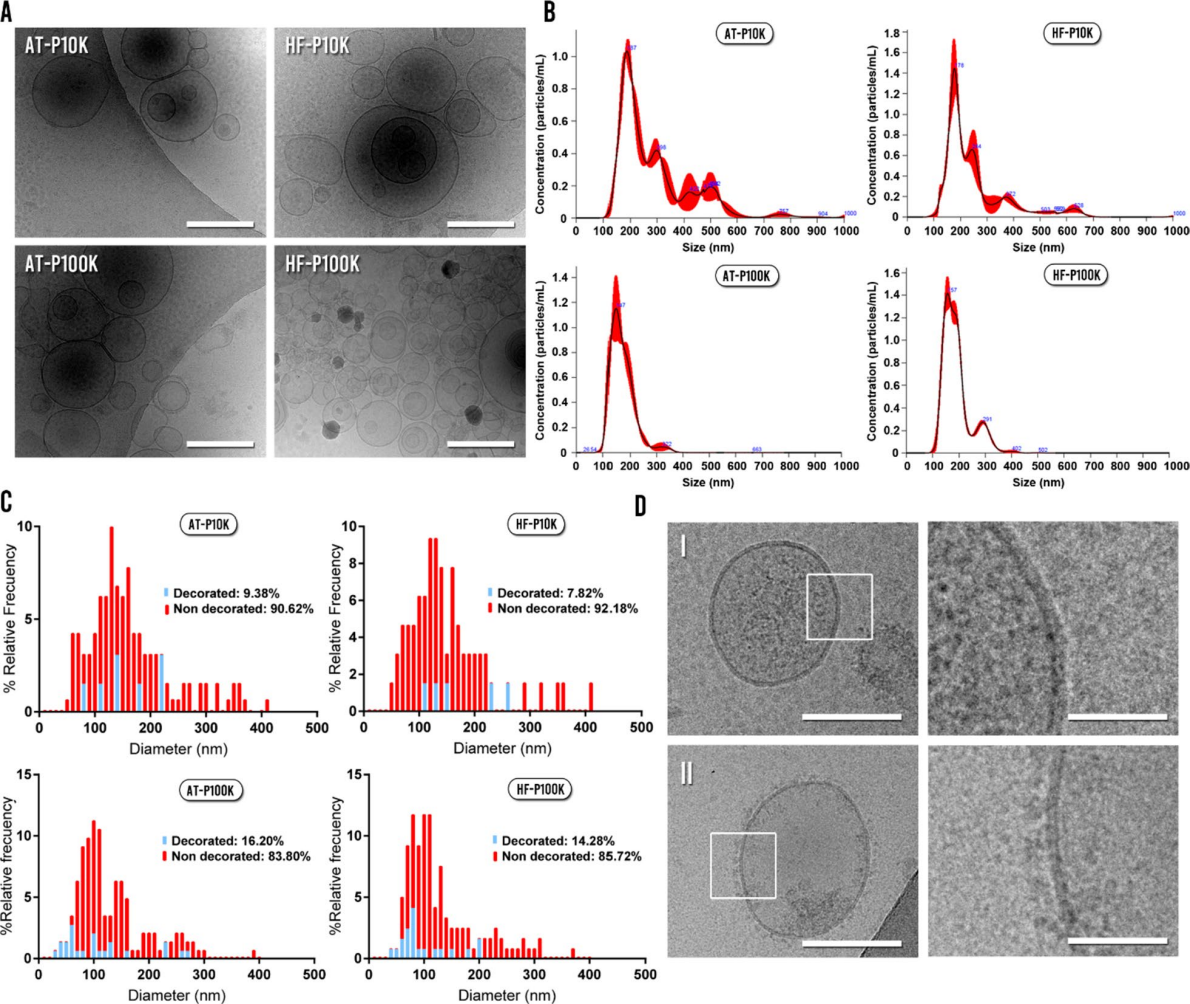
EVs size, morphology and protein decoration analysis. **a** Cryo-EM images of EVs. Scale bars are 200 nm. **b** NTA size-profile of the isolated fractions. **c** Size profile and protein decoration analysis of the different fractions calculated by measuring Cryo-EM images. **d** Images of a non-decorated EV (I) and decorated EV (II) with their respective zoomed insets (right). Scale bars are 100 nm (left-sided images) and 20 nm (right-sided images)

As represented in Fig. 2a, Cryo-EM images showed that the majority of the obtained material was round-shaped and bi-membrane-bounded vesicular units. The diverse heterogeneity of the different populations of EVs can be appreciated by the distinct electron dense cargos and protein decorated / non-decorated membranes presented. For both cell types, NTA measurements showed sizes ranging from 100 nm to nearly 400 nm for the P100K EVs, and from 100 to 800 nm for the P10K (Fig. 2b).

We also analyzed and compared the morphology, size and protein decoration of the isolated EVs by using the cryo-EM images. We studied approximately 400 single EVs and the majority of the observed units had the aforementioned round shape morphology for both cell types. Notably, we also found a small population of EVs with irregular morphologies (Additional file 1: Fig. S1)—multilayered vesicles, long tubular, etc.—. The size distribution of EVs from cryo-EM images revealed populations of smaller size—< 50 nm—than by NTA, obtaining a higher proportion of sEVs for both cell types (Fig. 2c). Moreover, when we analyzed the presence or absence of membrane protein decoration, we found that the majority of EVs were non-decorated for both cell types (Fig. 2c, d). The decoration percentages of m-lEVs were 9.38% and 7.82% for AT-EVs and HF-EVs respectively, whereas in the case of sEVs the percentages were 16.20% and 14.28% for AT-EVs and HF-EVs respectively. However, we did not observe differences in the diameter of decorated vs non-decorated EVs between each EVs population (Additional file 1: Fig. S2).

**Fig. 3:**
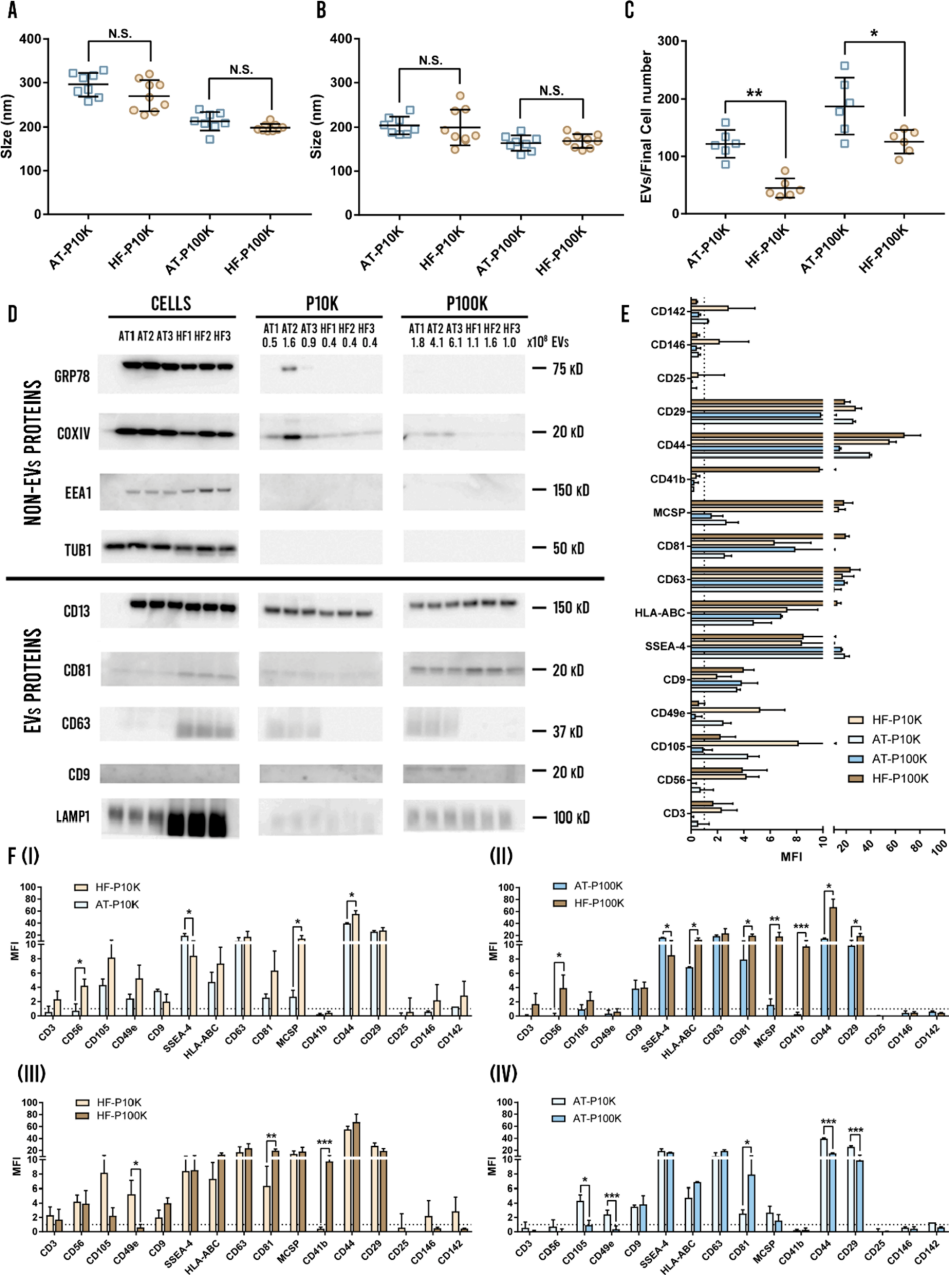
EVs batch-to batch analysis, western blotting and flow cytometry surface marker analysis. **a** Mean size of each EVs fraction for the different batches (*n* = 8). **b** Mode size of each EVs fraction for the different batches (*n* = 8). **c** Number of EVs compared to final cell count (*n* = 6). N.S. non-significant, *p < 0.05; **p < 0.005 between groups. **d** Western blotting analysis of EVs and non-EVs markers of three different batches for each cell type. **e** Multiplex bead-based flow cytometry assay for detection of EV surface markers (*n* = 3). **f** Comparison of EVs surface marker expression from (**e**): AT-P10K vs HF-P10K (I), AT-P100K vs HF-P100K (II), HF-P10K vs HF-P100K (III), and AT-P10K vs AT-P100K (IV). *p < 0.05; **p < 0.005; ***p < 0.001 between groups

When developing a EVs-based therapy it is crucial to achieve an adequate reproducibility in the batch-to-batch production. For such aim, we analyzed the mean and the mode size of the isolated EVs for the different obtained batches—every EVs isolation for each cell passage—. We did not find significant differences in any of the parameters for the different MSC-EVs (Fig. 3a, b). Furthermore, we obtained low relative standard deviations in the isolated batches for both cell types and pellets (Fig. 3a, b). As expected, we observed that the mean and mode sizes of EVs were lower for the P100K EVs than for the P10K EVs for both cell types. The overall deviations of the mean and mode size were also lower for the P100K compared to the P10K (Fig. 3a, b). In contrast, the efficiency of the obtained batches in terms of number of isolated EVs per final cell count showed significant differences between both cell types (Fig. 3c). Our results demonstrate that HF-MSCs produce less EVs than AT-MSCs for both P10K and P100K. Moreover, all cell viabilities at the end of every batch were adequate—between 95 and 98% for both cell types.

Marker analysis by western blotting (Fig. 3d) depicted the expression of CD13, CD81 and LAMP1 in P10K and P100K pellets derived from both cell types. In contrast, only AT-EVs showed bands for the rest of EV markers, with CD63 in both P10K and P100K, and CD9 in P100K. In the particular case of CD63, we observed different patterns; HF-MSCs retained this tetraspanin inside, while AT-MSCs showed it on m-lEVs and sEVs. We should mention that both MSC sources contain intracellular CD63 as exhibited by immunofluorescence images (Additional file 1: Fig. S3). We have also analyzed the presence of non-vesicular markers, including COXIV—a mitochondrial—and GRP78—endoplasmic reticulum—proteins, which appeared mostly in the P10K. Finally, TUB1, Hsp70, and EEA1 were only detected in the cells, and not in the vesicular fractions.

EVs were also evaluated by flow cytometry for the expression of 37 surface markers. We analyzed three different batches and found populations positive for CD3, CD9, CD25, CD29, CD41b, CD44, CD49e, CD56, CD63, CD81, CD105, CD142, CD146, MCSP, HLA-ABC/MHC-I and SSEA-4 (Fig. 3e). However, only CD9, CD29, CD44, CD63, CD81, CD105, MCSP, HLA-ABC and SSEA-4 were positive for all pellets and cell types. Nevertheless, we found some significant differences between both cell types and pellets. As observed, HF-P10K expressed CD56—not in AT-P10K—and more CD44 and MCSP than AT-P10K; in contrast, AT-P10K expressed more SSEA-4 (Fig. 3f-I). Regarding the P100K, we observed the same differences concerning the expression of CD44, CD56—not expressed in AT-P100K—, MCSP and SSEA-4 as in the P10K. Additionally, we achieved a higher expression of CD29, CD41b—not expressed in AT-P100K—, CD81 and HLA-ABC in the HF-P100K EVs (Fig. 3f-II). We also compared the P10K EVs and P100K EVs of the same cell type. As shown in Fig. 3f-III we found that P10K HF-EVs expressed more CD49b but lower CD81 and CD41b. On the other hand, P10K AT-EVs expressed more CD105, CD49e, CD44 and CD29 but lower CD81 than P100K AT-EVs (Fig. 3f-IV).

### EVs uptake and proliferation by human dermal fibroblasts

#### Uptake assay

**Fig. 4:**
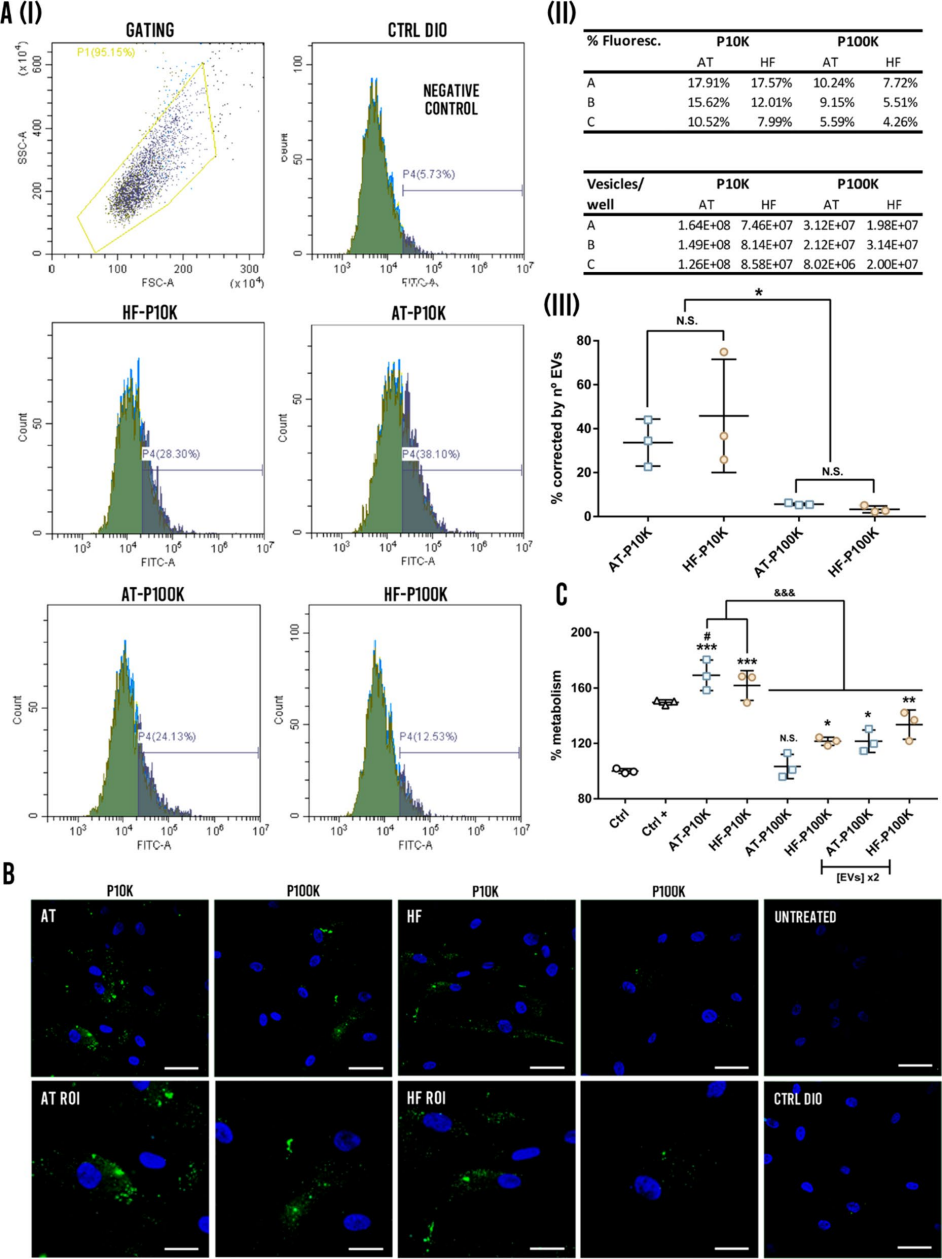
EVs uptake by HDFs and proliferation assay. **a** EVs flow cytometry uptake: (I) Gating of the cell populations and percentage of positive events for the background control (Ctrl DIO), and the EV preparations of 10 K and 100 K for HF and AT cells. (II) The upper table shows the percentage of fluorescent cells after adding different preparations of EVs. Bottom table shows the number of vesicles per well added for each preparation. (III) The graphs present the percentage of positive events according to the flow cytometry study corrected by the number of EVs for each type of EVs. *p < 0.05, N.S. Non-significance. **b** Representative fluorescence micrographs of the capture of EVs and region of interest (ROI) for each preparation. The figure at the bottom show HDFs treated with the negative control corresponding to the carry-over of fluorescence of cell culture media never conditioned with HDFs, and untreated HDFs. **c** HDFs proliferation assay by CCK8 (*n* = 3). N.S. Non-significant, *p < 0.05; **p < 0.005; ***p < 0.001 against the control group; ^#^p < 0.05 against the positive control group; ^&&&^p < 0.001 between groups

After incubating HDF cells with EVs coming from fluorescence-labelled AT- and HF-MSCs, we observed a comparable uptake of EVs coming from both types of MSCs (Fig. 4). Although there is a higher percentage of positive events corresponding to AT-EVs preparations (Fig. 4a-I, II), the correction by number of EVs in the preparation, together with the variability of obtained data, make those changes non-significant (Fig. 4a-III). However, there were almost 10 times more capture events when the cells are incubated with P10K preparations, corresponding to m-lEVs, than 100 K preparations (sEVs) (Fig. 4a-III and Additional file 1: Fig. S4). This difference can be observed directly by immunofluorescence (Fig. 4b). A very low carry-over of fluorescence was observed in medium that was not conditioned by HDFs (Fig. 4a, c).

#### Proliferation assay

It has been largely described that EVs promote the proliferation of different skin cell types such as HDFs among others [14]. When a chronic wound is given, the proliferative profile of the resident HDFs is impaired due to the impossibility to respond to the different cytokines and growth factors [24, 25]. In this regard, here, we compared the proliferative effect of the isolated MSC-EVs on HDFs. As shown in Fig. 4e, there is an important difference between the outcomes of P10K EVs as compared to the P100K EVs. P10K EVs showed an increase of approximately 60–70% of proliferation, as compared to the control, and AT-EVs, in addition, showed significant differences as compared to the positive control—complete medium—. In contrast, P100K EVs demonstrated a lower effect, which was dose-dependent. We did not notice significant differences between both cell types.

### Scratch assay

**Fig. 5:**
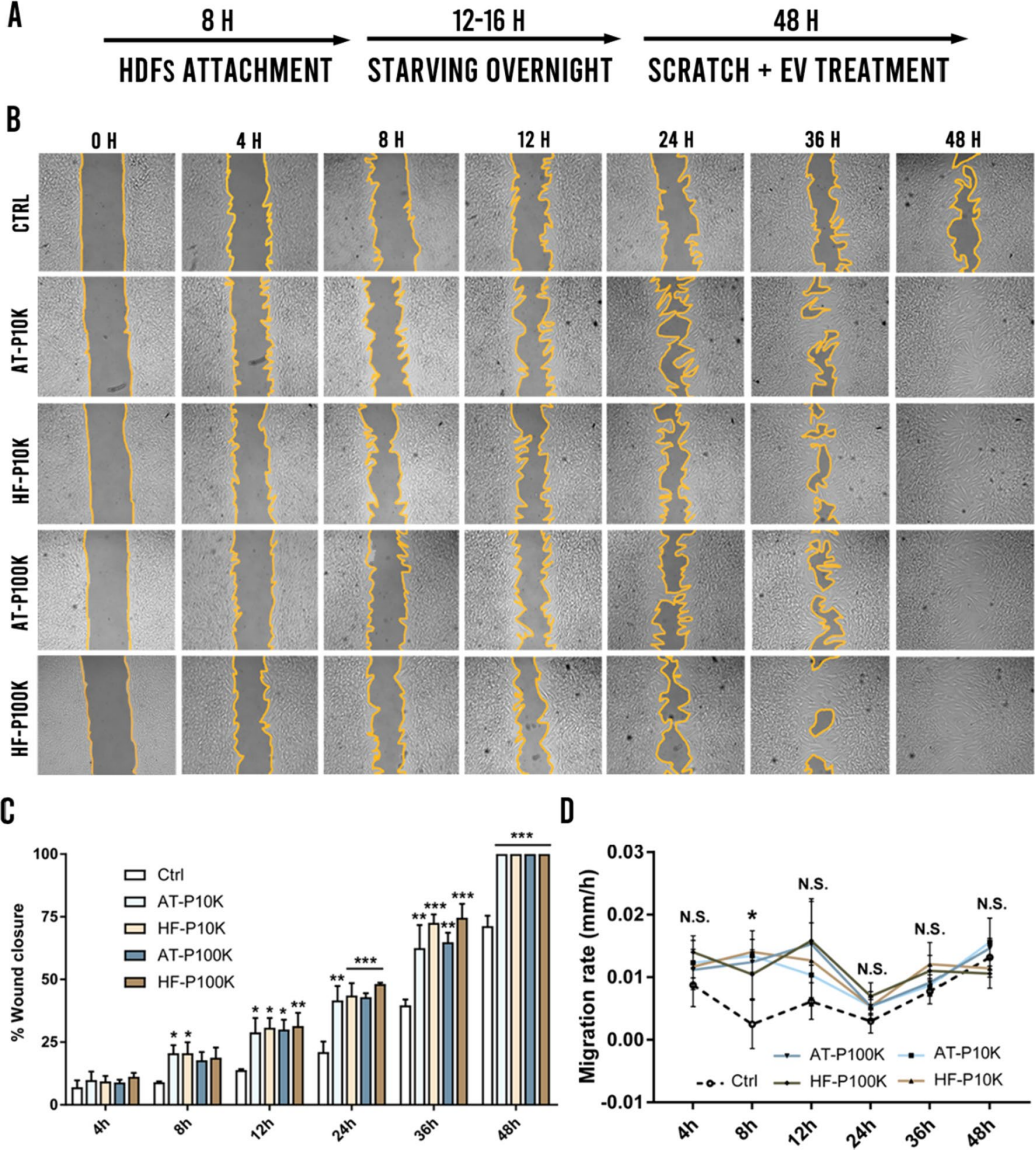
Scratch assay. **a** Experimental setup. **b** Images of the treated and control groups at different time-points of the scratch assay (*n* = 3). **c** Percentages of wound closure at the different time-points. *p < 0.05; **p < 0.005; ***p < 0.001 against the control group. **d** Migration rate of HDFs at different time-points. *p < 0.05 of all groups against the control group. N.S. Non-significant

In the scratch assay, we studied the pro-migratory effect of the MSC-EVs on HDFs. It is well-known that in chronic wound scenarios, there is also an impaired migration of these cells due to the harsh inflammatory environment and the impaired cell-to-cell communication [7, 24, 25]. In that sense, it is crucial to help these cells to increase their motility to favour the formation of granulation tissue ensuring an adequate closure of the wound. In this regard, we starved cells during the entire assay to minimize cell proliferation (Fig. 5a). Upon EVs treatment, we observed a faster wound closure of HDFs as compared to the control cells—treated with non-EVs control batches processed equally to the EVs batches—(Fig. 5b, c). A complete wound closure was achieved in all EVs-treated groups within 48 h, whereas the wound was still open in the control group (Fig. 5b). We did not observe significant differences between any of the EVs groups however, AT-P10K and HF-P10K obtained earlier significant differences—vs control group—as compared to their respective P100K EVs. We also measured the migration rate of the HDFs. As observed in the Fig. 5d only at the time-point of 8 h we achieved an increased migration rate in all groups as compared to the control group. Surprisingly, we did not obtain significant differences among all groups in the rest of the time-points. These results depicted that the main effect of EVs on the migration of HDFs was given between 4 and 8 h of treatment and maintained during the rest of the time-points.

### Tube formation assay

**Fig. 6:**
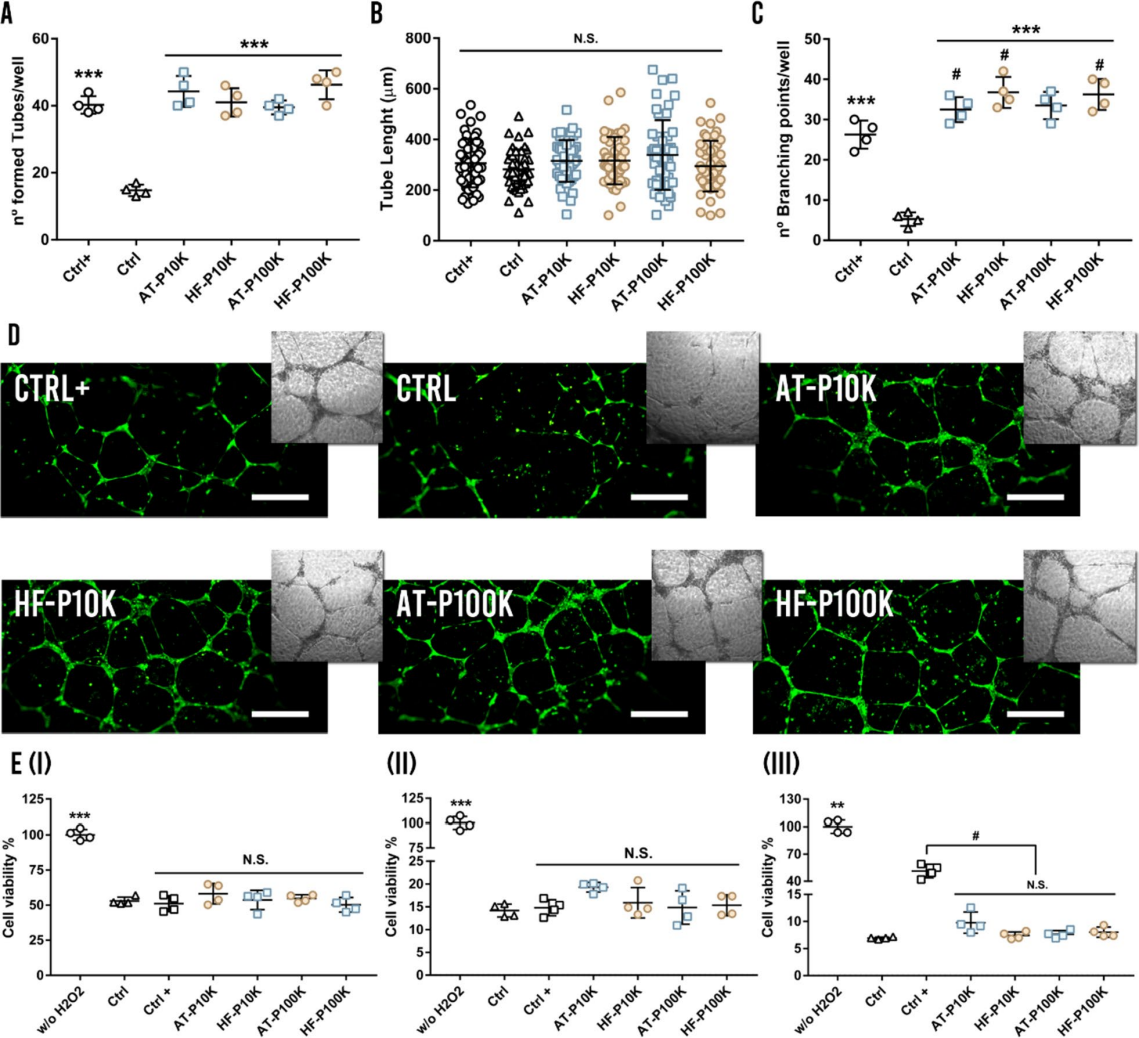
Angiogenesis and antioxidant assays. **a** Number of formed tubes per well (*n* = 4). **b** Diameter length of the formed tubes. **c** Number of branching points per well. ***p < 0.001 against the control group, ^#^ p < 0.05 against the positive control group, N.S. non-significance. **d** Calcein-stained fluorescence and brightfield images of the formed vessels. Scale bars are 500 µm. **e** Cell survival under a H_2_O_2_-mediated ROS environment (I) after a 2 h pre-treatment with EVs (*n* = 4), (II) after a 6 h pre-treatment with EVs (*n* = 4) and (III) under a 4 h co-treatment of cells with EVs and H_2_O_2_ (*n* = 4). **p < 0.005; ***p < 0.001, n.s. non significance against control group, ^#^ p < 0.05 between groups

The biological process of angiogenesis involves the proliferation, migration and tube formation of endothelial cells. The formation of new vessels can determine the outcome of chronic wound healing because the newly formed vessels allow the oxygenation and nutrient supply to the wound site [7, 26]. To analyze the effect of the studied MSC-EVs on migration and tube formation of endothelial cells, EVs were incubated with HUVECs on a Matrigel® niche. As shown in Fig. 6a, the groups treated with EVs remarkably enhanced the number of formed tubes as compared to the control group—HUVECs treated with non-EVs control batches processed equally to the EVs batches—. We did not obtain significant differences between the EVs-treated groups and the positive control group—complete medium—. Neither were differences found within the groups treated with EVs—neither P10K nor P100K—.

Furthermore, we studied the tube diameter length and the number of branching points—union points of formed tubes—(Fig. 6b, c). We did not observe differences in the tube diameter length between the different cell types, pellets and controls but, in accordance with the number of formed tubes, the treated groups obtained a higher number of branching points in comparison with the control group (Fig. 6d). Moreover, AT-P10K, HF-P10K and HF-P100K obtained a higher number of branching points as compared to the positive control.

### Reactive oxygen species (ROS) protection assay

Having achieved biological effects on HDFs proliferation and migration, we then studied the protective effects of EVs against ROS, which are commonly present in chronic wound scenarios [27]. As can be seen in the Fig. 6e, we did not obtain significant differences in the EVs-treated groups as compared to the control group in any of the performed assays. We observed that a 2 h and 6 h pre-treatment of HDFs with complete medium—positive control—did not showed differences in the ROS apoptotic protection as compared to the control group (Fig. 6e-I, II). However, in a 4 h of co-culture with EVs and H_2_O_2_, only the positive control achieved better outcomes as compared to the rest of the groups. This may be expected because of the presence of ascorbic acid in the medium supplementation (Fig. 6e-III).

### Cell survival under hyperglycaemia assay

**Fig. 7:**
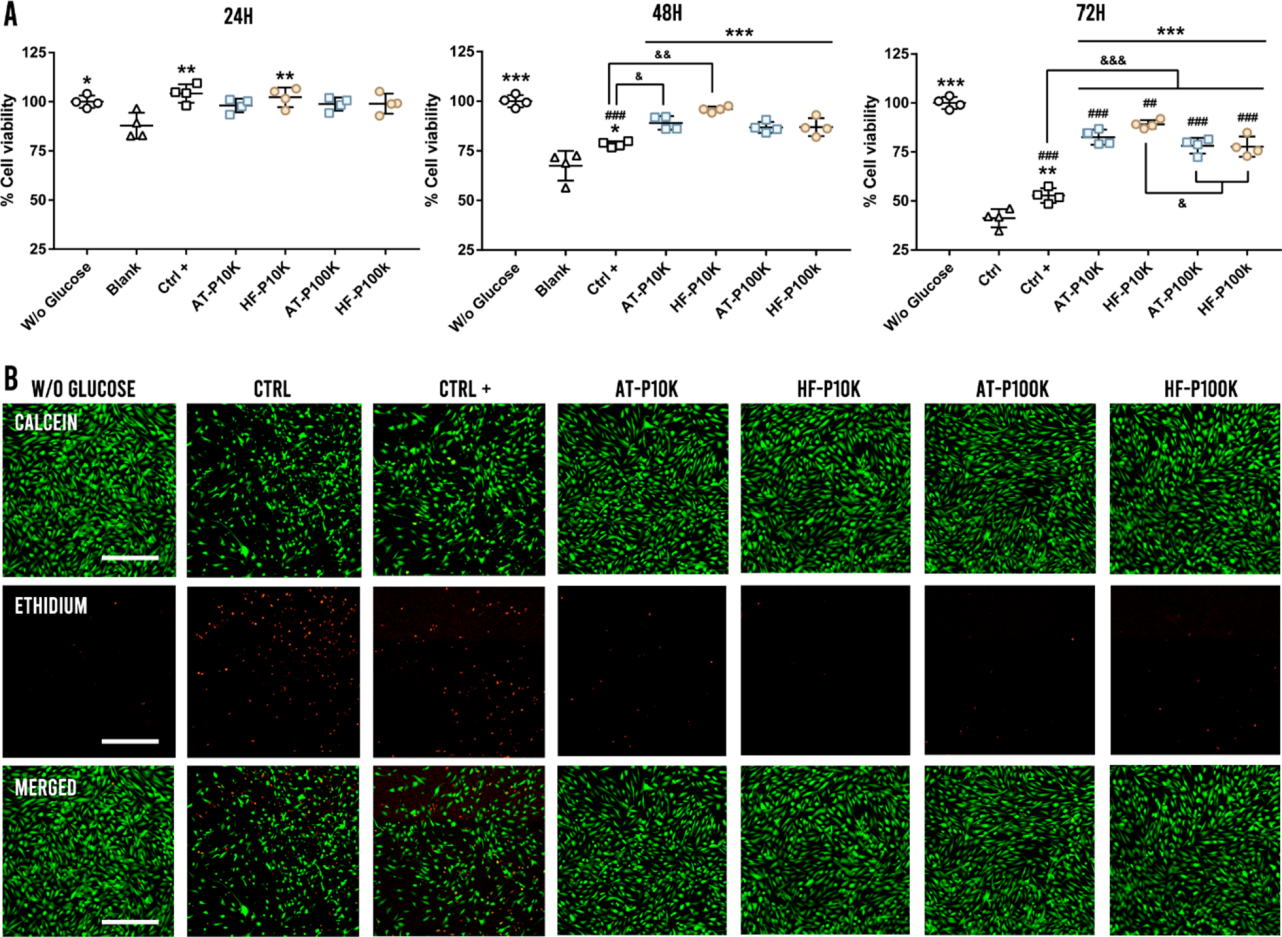
Cell survival under hyperglycemia assay. **a** Percentage of cell viability under hyperglycemia at 24 h, 48 h and 72 h (*n* = 4). *p < 0.05; **p < 0.005; ***p < 0.001 against the control group, ^##^ p < 0.005, ^###^ p < 0.001 against W/o glucose group; ^&^p < 0.05; ^&&^p < 0.005; ^&&&^p < 0.001 between groups. **b** Calcein/ethidium stained images of the survival cells at 72 h of hyperglycemia. Scale bars are 500 µm

Diabetic ulcers—a kind of chronic wounds—are characterized by a persistent inflammatory status with insufficient oxygen and nutrient accessibility caused by elevated glucose levels. As a result, a hypoxic environment with an elevated oxidative stress is formed, leading fibroblasts, as well as other skin cell types, to die [28, 29]. To study the cell survival of HDFs under a hyperglycemic environment we pre-treated HDFs with EVs for 6 h before exposing them to hyperglycemia. As observed in Fig. 7a, at 24 h we only obtained differences against the control group—HDFs treated with non-EVs control batches processed equally to the EVs batches—in the HF-P10K and positive control groups. However, after 48 h of hyperglycemic exposure, we achieved significant differences against the control in all the EVs-treated groups. Furthermore, AT-P10K and HF-P10K obtained higher outcomes as compared to the positive control group—complete medium—. We also observed that the EVs-treated groups did not obtain significant differences against the non-hyperglycemic group at this time point. Finally, at 72 h of hyperglycemia, we achieved significantly higher cell survival in the EVs-treated groups as compared to the control and positive control groups (Fig. 7b).

## Discussion

While MSC-EVs have gained the attention of scientific community in the field of tissue engineering and regenerative medicine, there is still a race for the development of the most suitable and effective MSC-EVs therapy. AT-EVs have largely demonstrated their potential in chronic wound healing scenarios [28, 30–36]. However, to the best of our knowledge, to date there is no work characterizing and analyzing the therapeutic potential of HF-EVs in this field, or in any other. For such aim, we first analyzed the size and morphology of the isolated EVs. Both AT-EVs and HF-EVs had round-shaped bi-membrane morphologies and the size ranges varied between 50 and 700 nm for P10K EVs and between 30 and 400 nm for P100K EVs by using NTA and Cryo-EM images. These size results are consistent with those reported in the literature using UC as the isolation method for MSC-EVs [37–40].

There is a vast number of works analyzing MSC-EVs by electron microscopy [41–44]. However, a few works have visualized MSC-EVs by using cryo-EM and none of them has quantified the percentage of their protein decoration [33, 45–47]. This technique allows reducing sample damaging and the appearance of artefacts caused by fixation, dehydratation or the addition of heavy metals [48]. Our results depicted that, by using UC as the isolation method, only a small percentage of MSC-EVs presented proteins decorating their membrane—approximately 8% on P10K EVs and 15% on P100K EVs—. Further studies should be carried out to analyze if the percentage of protein decoration on MSC-EVs would be modified by using different isolation/purification methods. However, a seminal work by Rizzo et al. described that, by using UC as the isolation method, the 90% of the isolated EVs—obtained from *C. Neoformans*—presented protein decorated membranes [49]. Moreover, another work by Noble et al. mentioned that m-lEVs had more protein-membrane decoration than sEVs in the MCF10A cell line by using cryo-EM images [50]. However, they did not quantify the percentage of membrane-decorated EVs. These results may suggest that the protein decoration of EVs could be more influenced by the EV-source rather than the biogenesis or isolation method used. In addition, it has also been described the spontaneous formation of a “protein corona” around the surface of nanoparticles, viruses and EVs as a protein aggregation on membranes [51–53]. Thus, a more thorough analysis should be made to identify if these molecules are spontaneously formed or native components of EVs, and whether they influence the biological activity of these EVs. For example, some authors have argued that the presence of these protein coronas on different nanoparticles have a major influence on their bioavailability and biodistribution [52, 53].

To ensure the reproducibility and quality of EV-based treatments, the establishment of a reliable and standardized isolation method for EVs is crucial. For such purpose, we studied the batch-to-batch reproducibility of the isolated EVs. Despite mean and mode size results had adequate reproducibility between batches for both pellets and cell types, results depicted that the number of EVs regarding the final cell count had greater variations. These results may suggest that AT-MSCs produce naturally larger quantities of EVs than HF-MSCs in the studied conditions. The characterization of the EVs protein markers by western blotting showed the presence of CD13—a marker of stemness associated to regeneration properties—in both cell lines [54], as well as the presence of CD81 in all the preparations. The results for CD63 presented a different trafficking of this molecule, since AT-MSCs released more protein in EVs, while HF-MSCs cells kept most of the protein intracellularly. This retention, together with the absence of CD9 in HF-EVs, the slightly increase of CD81 in the sEVs population secreted by HF-MSCs, and the retention of LAMP1 in HF-MSCs, suggest that both cell lines had a different balance of endosomal/ectosomal mechanism of EVs biogenesis [55]. Nevertheless, further studies inhibiting those routes would be necessary to clarify their relative importance. We also observed the presence of COXIV, a protein associated to mitochondria. The presence of mitochondrial proteins in some populations of EVs has been described in melanoma preparations [56], among other cell types, giving rise to the recently coined term “mitovesicles” [57].

The surface marker analysis of EVs by flow cytometry depicted significant differences in the expression profile of different proteins between both cell types and pellets. Using this technique, we demonstrated the presence of the transmembrane proteins CD9, CD63, and CD81 in all EVs. Interestingly, the results observed for those markers differ from western blot analysis. The reasons behind may lie in the technical differences between both approaches; bead capture only measures exposed epitopes, while western blotting shows the contribution for all the protein content of the preparation. Western blot quantification has also information regarding the size of the protein. Additional file 1: Fig. S5 shows the presence of bands detected by the antibody with higher strength in HF-EVs, below 37 kDa, which certainly can compensate the differences observed in the western blot between 37 and 50 kDa.

Another highly expressed marker in all of our EVs was SSEA-4, an embryonic stem cell marker popularly known as a “stemness” marker. It has been described the presence of this molecule in EVs from bone marrow MSCs (BM-MSCs), AT-MSCs [58, 59] as well as in other MSCs [60, 61]. Based on our findings, we observed the expression of SSEA-4 in sEVs and m-lEVs derived from both HF-MSCs and AT-MSCs. Furthermore, we also noticed the expression of CD105, a characteristic MSCs marker [62]. Specifically, we observed more expression in P10K EVs than in P100K EVs, for both cell types. In terms of functionality, AT-EVs and HF-EVs expressed several adhesion molecules such as CD29, CD44 and CD49e—mostly in P10K EVs—, which facilitate their homing to inflamed and injured tissues such as chronic wounds [63]. Another interesting marker found slightly represented in both P10K EVs—but not in P100K—was CD146 or melanoma cell adhesion molecule (MCAM), a molecule involved in permeability, monocyte transmigration and angiogenesis that is present in many tumors and endothelial cells [64, 65]. Another melanoma-associated marker found in all EVs—but especially found in HF-EVs—was MCSP. This molecule is a highly expressed marker in benign and malign melanomas and melanoma-derived EVs [66, 67]. However, the expression of MCSP in healthy tissues has also been described [66]. Ghali et al. demonstrated that HF-MSCs, among other hair follicle surrounding cells, express MCSP [68]. Thus, our results depicted that the EVs shuttled by HF-MSCs also express MCSP. Tissue factor (TF), also known as CD142 is recognized as one of the most important players in the coagulation process [69, 70]. Furthermore, it has been described that this marker also plays a role in wound re-epithelialization and necrosis [70]. The fact that principally P10K HF-EVs expressed this marker may suggests a more important role for HF-EVs than AT-EVs in the earliest phases of the wound healing process. Another receptor surprisingly found only in HF-P100K was CD41b or integrin αIIβ. This marker is a well-known collagen-binding integrin found in MSCs, as well as in many other cell types such as platelets. [71–73]. Finally, the expression of CD56 was exclusively found in HF-EVs. The presence of CD56 has been commonly found in NK cells and EVs—with immunomodulatory properties [74, 75]—and in neural lineage cells [76]. However, It has been also described the presence of CD56 in HFs and this has been related to the maintenance of the hair follicle immune privilege [76–78]. Thus, the role of CD56 in HF-EVs should be investigated to clarify whether the presence of this molecule in HF-EVs may be related to immunomodulatory properties.

When we analyzed the HDFs uptake of EVs, we found no differences in the P10K and P100K uptake between both cell types. On the other hand, we found that these cells were able to better internalize m-lEVs than sEVs. Indeed, we observed an almost tenfold fluorescence increase in HDFs after they captured m-lEVs as compared to sEVs. Although different aspects can influence this result, such as the number of membrane bound fluorochrome per vesicle in both m-lEV and sEVs, it is noteworthy that P10K EVs were able to induce a higher increase in cell proliferation than P100Ks in both cell types. More studies should be made in this regard to clarify whether these effects are related to a higher internalization or to intrinsic properties of the different EV populations. Furthermore, an additional analysis should be carried out in order the test the internalization efficacy of HDFs in the presence of other cell types such as phagocytes, keratinocytes etc. Regardless of that, our results confirmed that both m-lEVs and sEVs were able to increase proliferation of fibroblasts, as previously described in MSCs [35, 79]. Interestingly, we found that P10Ks were able to increase the proliferation of HDFs up to 25–40% more than P100Ks. It has been previously described the proliferative effect of m-lEVs on AT-MSCs [35], however, we have not found any work comparing the proliferative potency of sEVs vs m-lEVs in AT-MSCs neither in HF-MSCs. Promoting the migration of fibroblasts has also been an important requirement for every developing therapy intended for chronic wounds [7]. Besides the proliferative effects of MSC-EVs on different cell types, it has been also described that MSC-EVs can also promote the migration of fibroblasts, among other cell types [80]. In this work, we demonstrated that HF-EVs and AT-EVs alike were able to promote the migration of HDFs. Furthermore, we found that the effect observed on HDFs migration was achieved between 4–8 h—regardless of cell and pellet type—8 h and was maintained until the end of the assay.

Numerous studies have demonstrated that the oxidative stress increases tissue inflammation resulting in a stagnation of the wound healing process [81, 82]. In addition, a dysfunction of the vasculature, along with limited neovascularization and angiogenic processes, creates hypoxic conditions in chronic wounds [7, 83]. Some works have demonstrated that EVs derived from different cell populations can protect diverse cell types against ROS-induced apoptosis [84, 85]. In this regard, different MSC-EVs obtained from bone marrow or umbilical cord have demonstrated ROS-protective effects on cardiac stem cells and HaCaT cells [86, 87]. In contrast, only few researchers have described protective effects against ROS-induced apoptosis by means of AT-EVs on cardiomyocytes [88], NIH3T3 fibroblasts [28] and HDFs [89]. Here, we did not achieve such protective effects in any of the studied conditions with any of our pellet or cell types. In keeping with these results, Matsuoka et al. only observed a 10% of viability increase in the EVs-treated groups when compared to the control group. However, they did not employ a EVs-depleted medium in the assay. Based on all these findings, a more thorough analysis is warranted to clarify the role of MSC-EVs on the viability of HDFs under H_2_O_2_ oxidative stress.

It has been largely described the angiogenic potential of different MSC-EVs such as those produced by BM-MSCs, umbilical cord MSCs (UC-MSCs) or AT-MSCs, among others. Such effect has been attributed to the transfer of pro-angiogenic proteins—VEGF, PDGF, EGF, FGF etc.—and mi-RNAs including miR-31or miR-125 among others [90–92]. In this work, we found pro-angiogenic properties not only in AT-EVs but also in HF-EVs with both assayed pellets. Once again, we did not observe differences among groups in the size or morphology of the formed vessels. The need for increased blood flow and oxygenation reaches a high importance in diabetic chronic ulcers. The hyperglycemic microenvironment associated with diabetes challenges cell survival in diabetic wounds, causing oxidative stress and delaying the healing. For example, it has been described that hyperglycemia destabilizes HIF-1α and impairs its function at a concentration of 30 mM [28]. Our results depicted that with only a 6 h of pre-treatment, all EVs were able to protect and increase cell survival of HDFs under a cytotoxic hyperglycemic environment after 24 h of treatment and at least until 72 h of assay. Interestingly, our results were even better than those obtained by the positive control group—complete medium—. Here, we found that both pellets from AT-MSCs and HF-MSCs were able to reduce oxidative stress and increase HDFs metabolism and viability under a hyperglycemic condition.

## Conclusions

In conclusion, in this work, we describe for the first time to our knowledge, a complete characterization and functional comparison of m-lEVs and sEVs obtained from HF-MSCs against those obtained from AT-MSCs. Here, we made an in vitro screening of different MSC-EVs populations in different bio-relevant assays in order to analyze the efficacy of the formulations. We demonstrated the presence of the classical EV-markers among many others involved in biological processes not only in sEVs but also in m-lEVs. In this regard, HF-EVs showed a better “pro-regenerative” marker profile than AT-EVs for both m-lEVs and sEVs. We also analyzed the batch-to-batch production, the morphology and the protein decoration of these EVs depicting some differences between both cell types and pellets. Furthermore, we demonstrated that HF-EVs, equal than AT-EVs, were able to induce migration and proliferation in HDFs, as well as angiogenesis in HUVECs. However, we found that m-lEVs obtained better outcomes increasing HDFs proliferation. We did not observe protective effects against H_2_O_2_-induced oxidative stress in HDFs, but did prove that HF-EVs could protect HDFs from cell death and oxidative stress under hyperglycemic conditions to the same extent as AT-EVs. Altogether, we here postulate a promising source of EVs—not only sEVs but also m-lEVs—obtained from HF-MSCs with a great potential for chronic wound treatments and regenerative medicine therapies in general.

## Supplementary Information

**Additional file 1**. Supplementary Information.

## Data Availability

Raw data is available from the corresponding author upon reasonable request.

## Abbreviations

MSCs: Mesenchymal stromal cells
EVs: Extracellular vesicles
HFs: Hair follicles
AT: Adipose tissue
HDFs: Human dermal fibroblasts
HUVECs: Human umbilical vein endothelial cells
NTA: Nanoparticle tracking analysis
WB: Western blot
Cryo-EM: Cryo electron microscopy
DAPI: 2-(4-Amidinophenyl)-1 h-indole-6-carboxamidine
CCK8: Cell counting kit 8
ROS: Reactive oxygen species
