# Supplementary material for: Extracellular vesicles from hair follicle-derived mesenchymal stromal cells: isolation, characterization and therapeutic potential for chronic wound healing
Source: Stem Cell Res Ther. 2022 Apr 8;13:147. doi: 10.1186/s13287-022-02824-0 (PMC8994406; doi:10.1186/s13287-022-02824-0)
Supplement: Supplementary file 1 — Additional file 1. Supplementary Information. [file 13287_2022_2824_MOESM1_ESM.docx]

**Extracellular Vesicles from Hair Follicle-derived Mesenchymal Stromal Cells: Isolation, Characterization and Therapeutic Potential for Chronic Wound Healing**

Kevin Las Heras^1,2^, Félix Royo^3,4^, Clara Garcia-Vallicrosa^3^, Manoli Igartua^1,2,5^, Edorta Santos-Vizcaino^1,2,5^, Juan M Falcon-Perez*^,3,4,6^ and Rosa Maria Hernandez*^,1,2,5^

^1^NanoBioCel Group, Laboratory of Pharmaceutics, School of Pharmacy (UPV/EHU), 01006 Vitoria-Gasteiz, Spain.

^2^Bioaraba, NanoBioCel Research Group, Vitoria-Gasteiz, Spain.

^3^Center for Cooperative Research in Biosciences (CIC bioGUNE), Basque Research and Technology Alliance (BRTA), Exosomes Laboratory, 48160 Derio, Spain

^4^Centro de Investigación Biomédica en Red de Enfermedades Hepáticas y Digestivas (CIBERehd), 28029 Madrid, Spain

^5^Biomedical Research Networking Centre in Bioengineering, Biomaterials and Nanomedicine (CIBER-BBN), 28029 Madrid, Spain.

^6^IKERBASQUE, Basque Foundation for Science, 48013 Bilbao, Spain

* Rosa Maria Hernandez and Juan M. Falcon-Perez equally share credit for senior authorship.

****E-mail addresses*** [***rosa.hernandez@ehu.eus***](mailto:rosa.hernandez@ehu.eus) *and* ***jfalcon@cicbiogune.es***

**SUPPLEMENTARY INFORMATION**

**
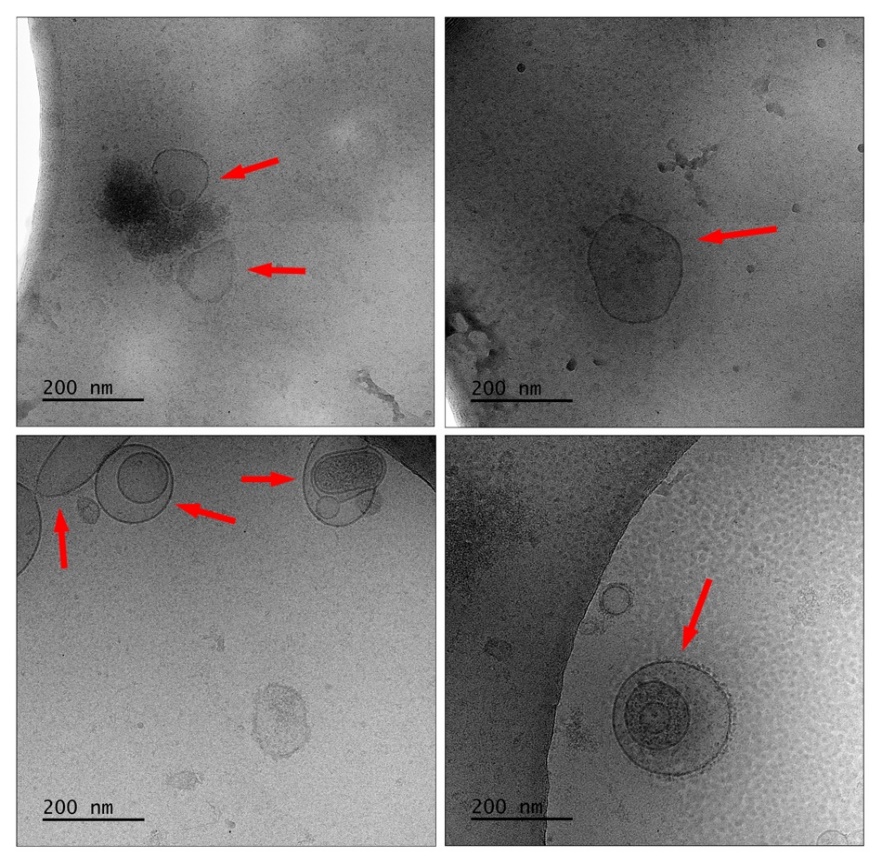
**

**Fig. S1. Cryo-EM images of the diverse heterogeneity of EVs populations**


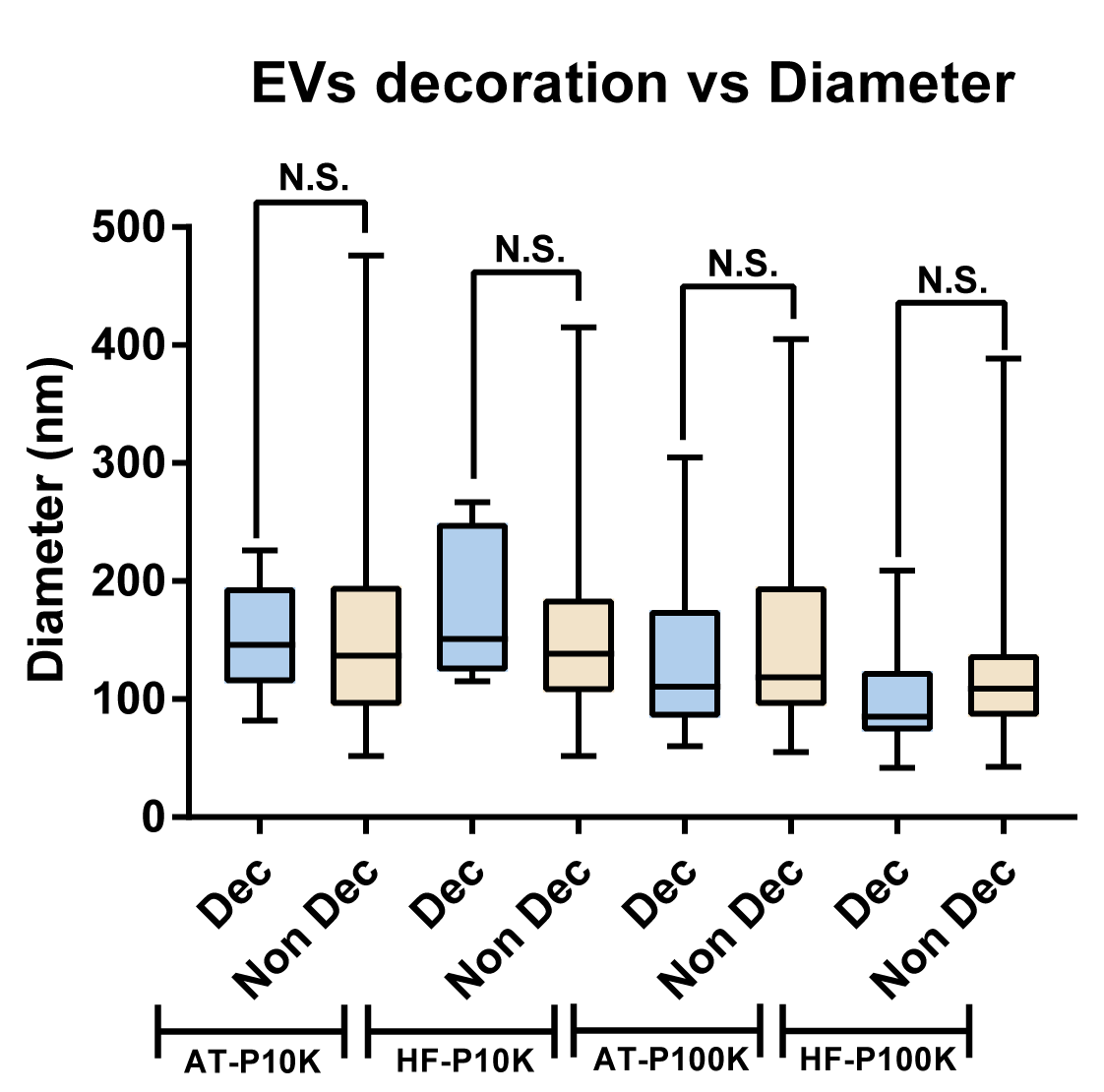


**Fig. S2. Comparison in decoration of EVs vs EVs diameter**

**
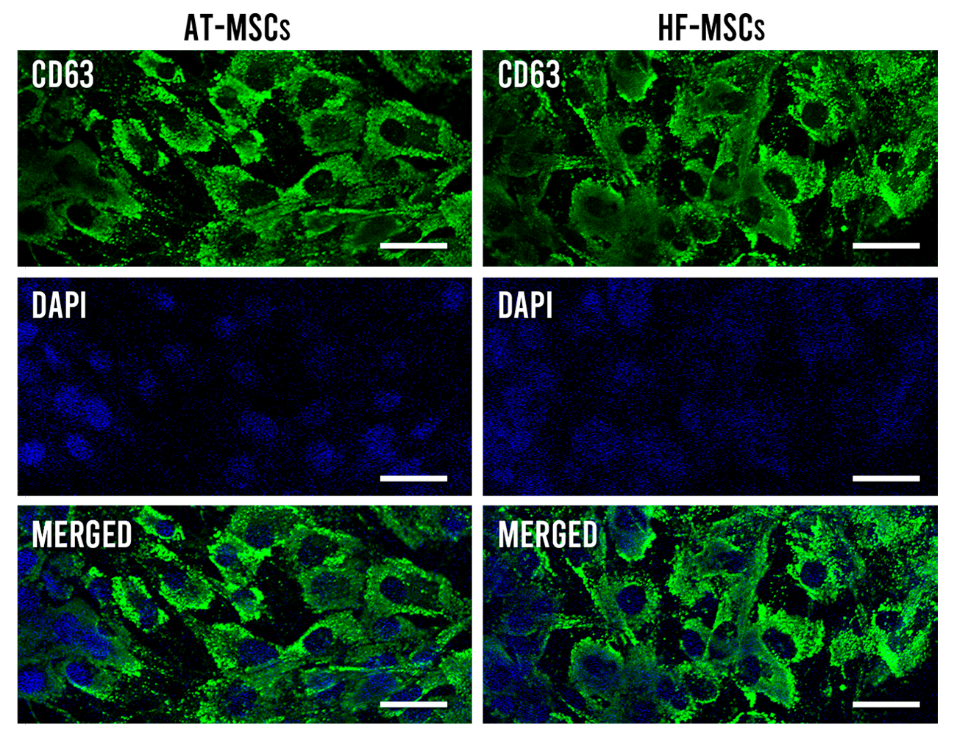
**

**Fig. S3. CD63/DAPI staining of AT-MSCs and HF-MSCs. Scale bars are 40 µm.**

**
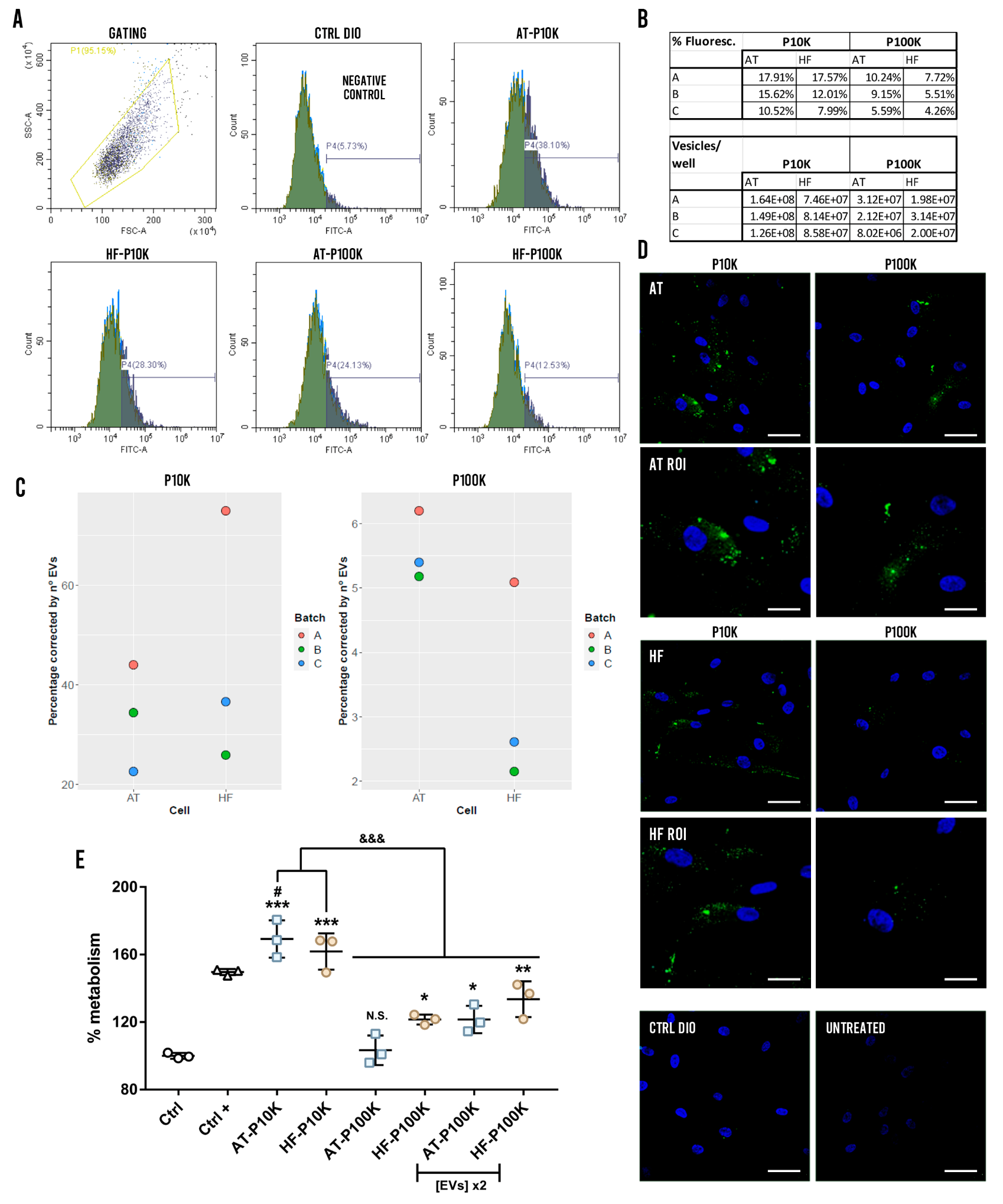
**

**Fig. S4. Percentage of positive events according to the flow cytometry study corrected by the number of EVs for each type of EVs and for the different batch preparations.**


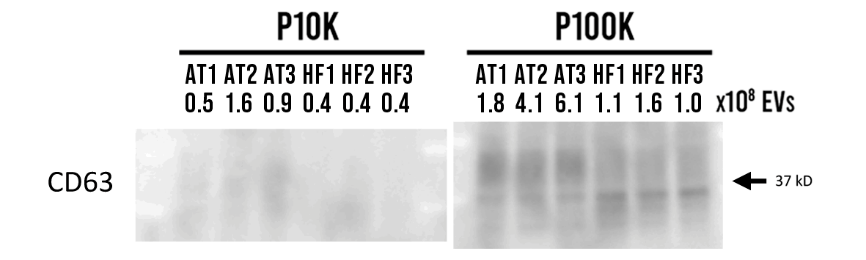


**Fig. S5. CD63 Western Blot extended membrane**
